# The Role of IL-33 in Gut Mucosal Inflammation

**DOI:** 10.1155/2013/608187

**Published:** 2013-05-23

**Authors:** Luca Pastorelli, Carlo De Salvo, Maurizio Vecchi, Theresa T. Pizarro

**Affiliations:** ^1^Department of Pathology, Case Western Reserve University School of Medicine, Cleveland, OH 44106, USA; ^2^Gastroenterology and Gastrointestinal Endoscopy Unit, IRCCS Policlinico San Donato, 20097 San Donato Milanese, Italy; ^3^Department of Biomedical Sciences for Health, University of Milan School of Medicine, 20122 Milan, Italy

## Abstract

Interleukin (IL)-33 is a recently identified cytokine belonging to the IL-1 family that is widely expressed throughout the body and has the ability to induce Th2 immune responses. In addition, IL-33 plays a key role in promoting host defenses against parasites through the expansion of a novel population of innate lymphoid cells. In recent years, a growing body of evidence has shown that the proinflammatory properties displayed by IL-33 are detrimental in several experimental models of inflammation; in others, however, IL-33 appears to have protective functions. In 2010, four different research groups consistently described the upregulation of IL-33 in patients with inflammatory bowel disease (IBD). Animal models of IBD were subsequently utilized in order to mechanistically determine the precise role of IL-33 in chronic intestinal inflammation, without, however, reaching conclusive evidence demonstrating whether IL-33 is pathogenic or protective. Indeed, data generated from these studies suggest that IL-33 may possess dichotomous functions, enhancing inflammatory responses on one hand and promoting epithelial integrity on the other. This review focuses on the available data regarding IL-33/ST2 in the physiological and inflammatory states of the gut in order to speculate on the possible roles of this novel IL-1 family member in intestinal inflammation.

## 1. Introduction

The intestinal epithelium is the largest surface area of the human body in direct contact with the external environment and exposed to a multitude of foreign microorganisms, macromolecules, and xenobiotics. As such, a fine regulation of gut mucosal immune functions is needed to develop a prompt and self-limiting inflammatory response against harmful pathogens but also to maintain normal gut homeostasis when no potential threat is sensed. Complex interactions between different cell types, effectors of both innate and adaptive immunity, regulate the inflammatory status within the intestinal mucosa. Pro- and anti-inflammatory cytokines represent key players in shaping this network and maintaining the communication among various cell types; balance among these mediators appears to be critical for gut immune homeostasis. In fact, a broad wealth of evidence demonstrates the importance of cytokine dysregulation in the onset of inflammatory conditions of the gastrointestinal tract. In particular, IBD, namely, Crohn's disease (CD) and ulcerative colitis (UC), is characterized by a significant dysregulation of cytokine production, with, in general, an overabundance of proinflammatory mediators [1]. For example, it has been shown that in the inflamed mucosa of IBD patients and colitis models, there is a perturbation of the balance between the proinflammatory cytokine, IL-1, and its naturally occurring antagonist, the IL-1 receptor antagonist (IL-1Ra), and that restoring this balance by exogenous administration of IL-1Ra ameliorates intestinal inflammation [2, 3]. As in the case of IL-1, other members of the IL-1 family, such as IL-18, have also been implicated in the initiation and perpetuation of chronic intestinal inflammation [4–6]. 

The IL-1 family of cytokines is constantly expanding, and, very recently, new members have been identified and studied, such as IL-1F11, IL-1F6/8/9, IL-1F7, and IL1F10, respectively known as IL-33, IL-36, IL-37, and IL-38 [7]. To date, of these novel members, IL-33 is the best characterized in terms of function and biological effects since its initial description in 2005 [8]. However, controversy still exists as to its precise role in intestinal disorders, particularly in the development of IBD. Thus, the aim of this review is to summarize what is already established regarding the role of IL-33 in the GI tract, while providing insight into the potential role of this novel IL-1 family member in the pathogenesis of chronic intestinal inflammation.

## 2. IL-33: A Novel Member of the IL-1 Family

 In 2003, a novel 30 kD protein, localized the nuclei of endothelial cells, was identified and shown to be highly expressed in high endothelial venules of tonsils, Peyer's patches, and lymph nodes [9]. The authors recognized, within the amino-terminal part of this molecule (aa 1–160), a novel homeodomain-like Helix-Turn-Helix (HTH) DNA-binding domain. As such, this protein was hypothesized to possess nuclear factor function, critical for the induction of a lymphatic endothelium phenotype, and was therefore coined “Nuclear Factor-High Endothelial Venules” (NF-HEV) [9]. Two years later, NF-HEV was identified as a novel member of the IL-1 family, shown to be the ligand for the former orphaned receptor, ST2, and renamed IL-1F11 or IL-33 [8]. In this first report, IL-33 was described as a potent enhancer of Th2 responses, inducing the production of IL-5 and IL-13. IL-33 was reported to be widely expressed in different cell types and within most organs throughout the body. In fact, IL-33 has been detected in cells of both hematopoietic origin, particularly in restricted populations of professional antigen presenting cells such as macrophages and dendritic cells, and in several different cell types of nonhematopoietic origin such as fibroblasts, adipocytes, smooth muscle cells, endothelial cells, bronchial and intestinal epithelial cells [8].

**Figure 1:**
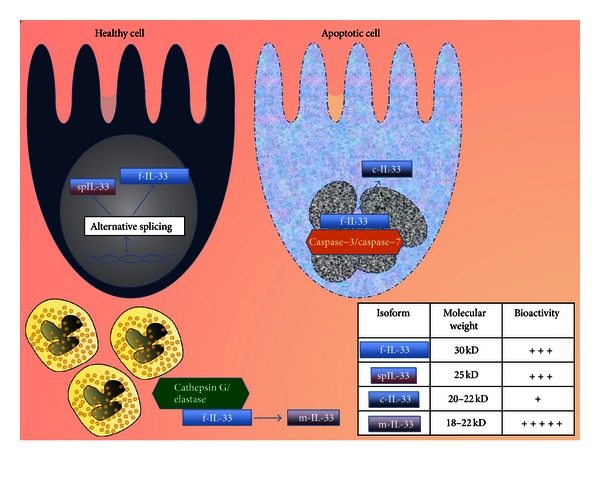
IL-33 isoforms and their associated bioactivity. IL-33 is synthesized as a 30 kD protein (full-length-IL-33, f-IL-33); however, alternative splicing can generate a 25 kD peptide (splice-IL-33, spIL-33), which possesses similar bioactivity to f-IL-33, but lacks caspase cleavage sites. During cellular apoptosis, f-IL-33 serves as a potential substrate for pro-apoptotic caspases (caspase-3 and caspase-7), generating smaller peptides of 20–22 kD with a marked reduction in bioactivity. Conversely, when secreted extracellularly, the proinflammatory activity of f-IL-33 may be potentiated in the context of a permissive, proinflammatory environment. In fact, Cathepsin G and elastase, released extracellularly by degranulating neutrophils, are able to cleave f-IL-33 into smaller isoforms (mature-IL-33, m-IL-33, 18–22 kD), which have been reported to display the greatest bioactivity.

Initially, it was thought that IL-33 was synthesized as a full-length 30 kD protein (full-length IL-33, f-IL-33) and processed by caspase-1 upon inflammasome activation, resulting in an alleged 18 kD bioactive peptide in a similar fashion to the other major IL-1 family members, such as IL-1β and IL-18 [8]. However, further investigation by three independent research groups revealed that the inflammasome paradigm for IL-33 did not occur in the *in vivo* setting and, instead, demonstrated that f-IL-33 possessed full bioactivity, while proapoptotic caspase-3 and -7 processed f-IL-33 into less bioactive forms of 20–22 kD (cleaved IL-33, c-IL-33) [10–12]. More recently, f-IL-33 has been shown to serve as a substrate for neutrophil elastase and cathepsin G, resulting in 18–22 kD peptides with an increased bioactivity of tenfold, suggesting a possible extracellular mechanism to amplify the effects of IL-33 during inflammatory conditions [13]. To add further complexity to this scenario, an alternative splice variant of IL-33 (spIL-33) has been described that is 5 kD smaller than f-IL-33 and lacks the exons cleavable by caspases, but possesses similar bioactivity to f-IL-33 [14].  Figure 1 summarizes the current knowledge regarding the different IL-33 isoforms/splice variants and their bioactivity. 

As previously mentioned, IL-33 exerts its biological effects through the binding of its receptor, ST2, also known as IL-1 receptor-like 1 (IL1RL1), belonging to the Toll-IL-1 Receptor (TIR) superfamily [8]. ST2 exists in two different splice variants, leading to the synthesis of proteins with opposite biological functions: ST2L, a transmembrane receptor that activates downstream signaling upon IL-33 recognition, and sST2, a soluble molecule that likely serves as a decoy receptor by binding IL-33 and decreasing its availability to ST2L, the IL-33 signaling receptor [15]. Similar to other TIR receptors, ST2L requires pairing to a coreceptor in order to initiate the downstream cell signaling cascade. As such, the IL-33 receptor complex consists of ST2L and the IL-1 receptor accessory protein (IL1RAcP), a TIR member also involved in IL-18 signaling [16]. ST2 and IL1RAcP interact through their TIR domain with MyD88, TRAF6, and IRAK1/4, eventually leading to the activation of transcription factors, such as NF-*κ*B and AP-1, which promote the production of several proinflammatory mediators [8]. Another IL-1 family coreceptor member, that is, “single Ig IL-1R-related molecule” (SIGIRR) or Tir8, can also dimerize with ST2 and likely acts as a negative regulator of the IL-33/ST2 signaling pathway, ultimately reducing IL-33's biological effects [17]. To date, a very limited amount of information is available regarding the biologic and pathophysiologic relevance of IL-33 isoforms/splice variants, ST2 splice variants, and alternative ST2/SIGIRR signaling. 

## 3. IL-33 Is a Key Player in Mucosal Immunity against Intestinal Parasites

Since its first description, IL-33 has been reported to be localized in barrier epithelia within organs/tissues in direct contact with the external environment, including the skin, airway, and gut epithelia, suggesting a possible role of this cytokine in early immune responses against invasive pathogens [8]. Moreover, several studies consistently show that normal mice injected with recombinant IL-33 develop a marked epithelial cell hyperplasia in the pulmonary and GI tracts, together with an eosinophilic and mononuclear infiltration into the lamina propria, specifically localized in these barrier organs/tissues [18, 19]. Interestingly, the production of a thick mucus layer is one effective mechanism aimed to enhance epithelial barrier function and infers protection of these mucosal organs. It is commonly thought that these specific effects on intestinal epithelial cells are mediated by the Th2 cytokine IL-13 [20, 21], which is abundantly overexpressed after IL-33 stimulation/administration [8]; however, the possibility that IL-33 *per se* promotes epithelial differentiation towards a secretory type it may not be ruled out. In fact, IL-33 is also a potent inducer of Th2 cytokines that are pivotal in mounting potent immune responses against helminthes and fungi; in fact, early papers exploring IL-33 function have pointed out its fundamental role in eliminating intestinal parasites. For example, *Trichuris muris*, a nematode capable of infesting the GI tract, induces the production of high levels of IL-33 from the infected ceca of experimental animals [18]. In this experimental setting, the administration of IL-33 was shown to boost parasite clearance from host mice. Even though a significant increase of NK cells was detected in mesenteric lymph nodes (MLNs) from SCID and wild-type animals treated with IL-33, parasite clearance appeared to be mediated through T- and B-cell activation, as SCID mice failed to eliminate *T. muris* upon IL-33 administration [18]. IL-33 appeared to have similar protective functions, enhancing host responses against other parasitic and bacterial threats, such as *Toxoplasma gondii* [22], *Pseudomonas aeruginosa* [23], and* Leptospira* [24] infections, in different organ systems.

More recently, a growing body of evidence has shown that IL-33's protective effects against parasites are also mediated by a newly identified innate immune cell population, uniquely coined “nuocytes,” named after “nu,” the 13th letter of the Greek alphabet, making reference to their ability to produce high levels of IL-13 [19]. In fact, these innate effector leukocytes display unique phenotypic characteristics and are not aligned with any other known mature leukocyte population. They express ICOS, CD45, ST2, and IL-17BR and respond to IL-33 and/or IL-25 stimulation with a significant increase in IL-13 expression [19]. In addition, the transcription factor, ROR*α*, has been described to be necessary for their development [25]. Nuocytes appear to be early initiators of Th2 responses, and their activation is pivotal in eliciting worm clearance after *Nippostrongylus brasiliensis* infection, but a Th2 response is not required for nuocyte activity, as their development occurs in Th2 cytokine deficient mice and in RAG2 knockout mice [19, 26]. At the same time, similar IL-33/IL-25-responsive innate effector cell populations have been described by others. Moro et al. characterized a population, namely, natural helper cells (NHC), identified by the presence of cell surface ST2, c-Kit, Sca-1, and IL-7R, that reside in mesenteric adipose tissue and are organized in fat-associated lymphoid clusters [27]. Price et al. identified similar cells, but not expressing either Sca-1 or c-Kit, which were coined innate helper 2 (Ih2) cells and that are widely distributed in mouse MLN, spleen, liver, and bone marrow [28]. Together, these novel cell populations and others (e.g., multipotent progenitor type 2 (MPP^type  2^) cells [29]) are termed innate lymphoid cells (ILCs) and share significant biologic similarities. As such, it may be hypothesized that ILCs represent different maturation steps or different differentiation phenotypes from the same hematopoietic lineage. Nonetheless, nuocytes, NHC, and Ih2 cells are induced by IL-33 and are pivotal in mounting effective immune responses against helminthes characterized by the overproduction of IL-5 and IL-13 and the induction of histopathologic changes in the gut mucosa, including epithelial/goblet cell hyperplasia and eosinophilic infiltration. Whether or not these novel innate cell populations are the only responsible for the induction of these pathologic features is still debatable, as it has been clearly shown that IL-33 induces the production of chemokines for eosinophils [30] and may also directly affect epithelial cell biology. It seems reasonable that during parasitic infestation, in order to induce a prompt innate response, cells constituting intestinal barrier, such as epithelial cells and macrophages/dendritic cells, act as a major source of IL-33/IL-25; however, at present, no experimental data are available to confirm this hypothesis; novel experimental tools may provide important insights to this topic; in fact, mice genetically engineered to have the β-galactosidase-neomycin resistance fusion gene inserted in IL-33 intron 1, the IL-33-*LacZ* gene trap reporter mice, were recently described and used to specifically measure and localize IL-33 promoter activity within mouse body; indeed, these animals may be a valuable tool to identify the cellular sources of IL-33 during health and disease conditions, such as parasitic infestations. As a growing body of evidence confirms the importance of IL-33-induced ILCs in the protection against parasites, to date, the potential role of these novel cells in spontaneous inflammatory conditions has not been fully characterized in models of intestinal inflammation. 

## 4. IL-33 and ST2 Are Dysregulated in IBD

IL-1 family members, coordinating early innate responses and later adaptive immune responses, have been shown to play an important role in the pathogenesis of chronic intestinal inflammation, characterizing IBD [31, 32]. As such, IL-33 appeared to be a promising candidate to be studied in the setting of human IBD. In fact, in 2010, four independent groups described the dysregulation of IL-33 expression in patients with UC and to a lesser extent, CD [33–36]. Consistently, all groups showed increased protein levels of IL-33 within the inflamed mucosa of IBD patients compared to healthy controls, particularly in UC [33–36]. Immunohistochemistry experiments revealed intense IL-33 staining in lamina propria inflammatory infiltrates, primarily localizing in cells that morphologically resemble macrophages and B cells/plasma cells [35]. Importantly, nonhematopoietic cell types also contribute to the augmented production of IL-33 during intestinal inflammation; in particular, intestinal epithelial cells [33, 35, 36] and myofibroblasts [34] display the highest levels of IL-33 during active IBD. IL-33 was also detected in other cell types within gut mucosa, confirming previous reports in other organ systems, with expression in fibroblasts, smooth muscle cells, endothelial cells [8, 37], and adipocytes [38]. IL-33 was also detectable in the sera of IBD patients, with concentrations significantly increased compared to healthy controls [33, 35]. Circulating IL-33 levels were also found to be markedly reduced upon anti-TNF administration (Infliximab/Remicade) and may have the potential to be used as a marker of disease activity and/or response to anti-TNF treatment [35]. Intriguingly, IL-33 isoforms vary according to the site wherein they are detected. In fact, f-IL-33 was the only form found to be present in both the cytoplasm and nuclei of IL-33-producing cells, such as intestinal epithelial cells, whereas evaluation of mucosal biopsies revealed the presence of both f-IL-33 and 20–22 kD cleaved forms; conversely, sera displayed exclusively the presence of the cleaved 20–22 kD forms [35]. Taken together, these data suggest the presence of extracellular proteases that have the ability to cleave IL-33, possibly modulating its bioactivity. As mentioned earlier, neutrophil elastase and cathepsin G are capable of cleaving f-IL-33, generating more potent forms [13]. As such, the inflammatory milieu characterizing IBD may have the potential to amplify IL-33's biological effects. On the other hand, similar to proapoptotic caspases, extracellular proteases may instead have the ability to inactivate f-IL-33, perhaps in an attempt to prevent possible harmful effects that may be triggered by high circulating levels of this cytokine. Indeed, further data are needed in order to clarify the significance of the circulating forms of IL-33 and the mechanism(s) leading to their generation. 

Data regarding the analysis of IL-33 expression in human IBD were closely recapitulated in SAMP1/YitFc (SAMP) mice [35], a spontaneous model of chronic intestinal inflammation immunologically characterized by an early Th1 response and a later mixed Th1/Th2 phenotype [39], both significantly contributing to the extent of disease severity in these mice [40, 41]. In this study, SAMP mice were shown to display high IL-33 levels in the serum as well as the gut mucosa, consistently localized to intestinal epithelial cells and macrophages within the lamina propria. Interestingly, mucosal expression of IL-33 was found to positively correlate with the severity of SAMP enteritis [35]. 

 A substantial alteration of ST2 expression as well was detected in the intestinal mucosa and sera from IBD patients. ST2 was abundantly expressed in the inflamed mucosa of IBD patients compared to healthy controls, and similarly, elevated circulating levels of sST2 were shown in UC and CD patients [33, 35], correlating with mucosal ST2 expression and both clinical and endoscopic disease activities [42]. Indeed, this interesting piece of data may suggest that serum IL-33/sST2 is produced within intestinal mucosa and directly reflects the severity of mucosal inflammation; as such, it would be worthwhile to investigate the role of circulating IL-33 and sST2 as markers of disease. Besides quantitative differences of ST2 expression, striking qualitative alterations were detected in the inflamed IBD mucosa versus healthy tissues. ST2 was constitutively expressed by intestinal epithelial cells during normal conditions; however, in chronically inflamed IBD mucosa, intestinal epithelial cells lose ST2 expression, which is redistributed to other inflammatory cell types. Specifically, intestinal epithelial cells of UC patients did not present ST2, whereas ST2 localized to lamina propria professional antigen presenting cells and T helper lymphocytes [35]. If a robust increase of ST2-positive cells within the lamina propria infiltrate is a common feature of different inflammatory conditions of the gut, the epithelial reduction/disappearance of ST2 appears to be specific for IBD. In fact, in nonspecific colitides, such as infectious colitis and diverticulitis, ST2 appears to be upregulated in both the epithelial and immune compartments of gut mucosa [35]. Of note, epithelial dysregulation of ST2 refers to a marked decrease of ST2L, the IL-33 transmembrane receptor, but not of the sST2 protein [33, 35]. As such, this particular pattern of expression may suggest that during IBD, a severe impairment of IL-33 signaling within the epithelial layer occurs, whereas IL-33/ST2 engagement may be enhanced in intestinal immune cells. However, whether this alteration of epithelial ST2L is a feedback response to the chronic exposure of elevated IL-33 concentrations or an intrinsic epithelial defect characterizing IBD has yet to be determined.

Of note, during active IBD, an intense ST2 signal is detectable in perivisceral adipose tissue, where a rich infiltrate of ST2-positive immune cells is evident [35]. Consistent with recent data in the literature, this particular ST2-expressing cell population, dispersed within mesenteric fat, may represent the NHC population, recently described by Moro et al. [27], that are pivotal for the onset of immune responses against parasites, but whose possible role in idiopathic intestinal inflammation has not yet been explored.

Remarkably, as a further confirmation of the dysregulation of IL-33 and ST2 in human IBD, recent data, obtained in an Italian cohort of adult and pediatric UC and CD patients, demonstrate that specific IL-33 and ST2 gene polymorphisms confer an increased risk of developing IBD (both UC and CD), suggesting the involvement of the IL-33/ST2 axis in the onset of chronic intestinal inflammation [43].

## 5. The IL-33/ST2 Axis Exerts Dichotomous Functions during Idiopathic Intestinal Inflammation 

Despite robust data describing the changes in patterns of the expression of IL-33 and ST2, current data regarding the role of this novel cytokine/receptor pair in the onset of IBD is conflicting and scarce. In fact, while some studies suggest a proinflammatory function, others indicate a protective, anti-inflammatory role.

Since its first description as a cytokine, IL-33 has been shown to possess potent proinflammatory activity, inducing Th2 cytokine production and promoting Th2 immunity. Thus, IL-33 was initially identified as a possible target for dampening inflammation in several animal models of inflammatory diseases, such as airway inflammation and arthritis (i.e., ovalbumin challenge-induced airways inflammation and collagen-induced arthritis) [44–46] since it was well established that IL-33 had the ability to promote inflammation through both the recruitment [47] and activation [48] of immune cells to the site of inflammation, as demonstrated by data obtained in a Th2 cell adoptive transfer model and *in vitro*. Consequently, mice overexpressing IL-33 were reported to display spontaneous airway and lung inflammation [49], whereas blocking the IL-33/ST2 axis decreased inflammation in an experimental murine model of asthma [44]. Similarly, antagonizing IL-33's biological effects, using anti-ST2 blocking antibodies, was effective in ameliorating joint inflammation in a murine model of rheumatoid arthritis [46]; in fact, the administration of IL-33 to cultures of immune cells isolated from murine inflamed joints led to a dramatic increase in IL-5, IL-6, and IL-17 production [45, 46]. These trends were also observed in a chronic model of intestinal inflammation [35]. Unfractionated MLN cells, collected from inflamed SAMP mice, secrete high levels of the aforementioned cytokines when cultured in the presence of IL-33 [35]. Of note, both IL-5 and IL-6 have been shown to play a pathogenic role in SAMP ileitis, as demonstrated by the amelioration of intestinal disease following either anti-IL-5 or anti-IL-6 treatment [50, 51], whereas IL-17 has been extensively characterized as a key cytokine in many immune-mediated diseases, including IBD [52–54]. Along these same lines, unpublished data generated in our laboratory showed that blockade of the IL-33/ST2 axis significantly reduces intestinal inflammation in SAMP mice [55–57]. Mechanistically, this effect appeared to be associated with a decrease of lamina propria eosinophil infiltration, by downregulating IL-5 and eotaxin-1 and eotaxin-2 [57], and by reducing the percentages of IL-17 producing macrophages [55], a population that has previously been described in intestinal and airway inflammatory conditions [58, 59]. However, a full phenotypic and functional characterization of this nonclassical cell population has not yet been performed. In addition, IL-33-induced inflammation may play a role in the development of intestinal fibrosis, as SAMP mice treated with anti-ST2 blocking antibodies show decreased collagen deposition within the intestinal wall, together with a reduced production of pro-fibrotic molecules, such as transforming growth factor (TGF)-β, connective tissue growth factor (CTGF), collagen-1, insulin growth factor (IGF)-1, and matrix metalloproteinase (MMP)-9 [56]. Consistently, healthy AKR mice treated for one week with intraperitoneal injections of recombinant IL-33 developed a marked thickening of muscularis layer of the intestinal wall, which was accompanied by increased expression of collagen-1, collagen-3, IGF-1, and CTGF [56]. These data, along with the observation that *in vitro* IL-33 stimulation of human subepithelial myofibroblasts (SEMF) induces the expression of profibrogenic genes such as *col1a1*, *col3a1*, *ctgf*, and *tgfb1* [56], suggest that IL-33 may also play an important role in promoting inflammation-associated gut fibrosis, as reviewed by Lopetuso et al. [60]. 

Additional data, primarily obtained from chemically induced models of intestinal inflammation, provide further insight into the role of IL-33 and ST2 in gut inflammatory conditions. Oboki et al. induced colitis in IL-33 knockout (KO) mice and wild-type littermates using dextran sodium sulfate (DSS) administration [61]. The colonic inflammation caused by DSS is primarily initiated by disruption of the epithelial barrier, which results in bacterial translocation into the underlying lamina propria. In this model, the resulting inflammation is mediated by the activation of innate immune responses and occurs in a T-cell-independent manner [62]. During the acute phase of this experimental model, IL-33 KO mice presented with reduced histologic inflammatory scores, with a significant reduction of granulocyte infiltration when compared to wild-type mice [61]. Other independent groups replicated similar results, using the same acute animal model [63–65]; in addition, Sedhom et al. confirmed the pathogenic role of IL-33 also in a different model of intestinal inflammation, the trinitrobenzene-sulfonic-acid- (TNBS-) induced colitis, which is obtained throughout the chemical haptenization of protein expressed within the gut wall [64]. Interestingly, Sedhom et al. demonstrated on both DSS- and TNBS-induced colitis that, during the onset of intestinal inflammation, the IL-33/ST2 axis activation was able to affect the non-haematopoietic component of the inflammatory response, resulting in a significant impairment of the epithelial barrier function [64]. Conversely, results obtained by Oboki et al., during the recovery phase of the DSS-induced colitis in IL-33 KO mice, suggest that IL-33 might have different roles during different phases of the inflammatory process; in fact, weight recovery was markedly delayed in IL-33 KO mice, with a slight increase in mortality rate [61]. These results suggest that IL-33 is a critical amplifier of innate immune responses within the gut mucosa, whereas its role in the maintenance of chronic inflammation is less clear. Alternatively, IL-33 may possess an important functional role in enhancing innate immune responses related to bacterial clearance or in promoting mucosal wound healing.

Despite its well-known proinflammatory properties, IL-33 has also shown protective functions in different diseases; in fact, early reports demonstrated that IL-33 exerts a cardioprotective effect, reducing overload-induced cardiomyocyte hypertrophy [66]; on the same line, Miller et al. showed that IL-33 reduced the development of atherosclerosis [37] and the inflammation within the adipose tissue of obese mice [67], confirming the beneficial effect of IL-33 on the cardiovascular system. In addition, IL-33 appeared to have some protective role on various inflammatory conditions as well. For example, IL-33 appears to reduce inflammation in Con-A hepatitis [68], in central nervous system demyelinating disorders [69], and in pancreatitis [70]. Moreover, the ability of IL-33 to recruit neutrophils to the site of inflammation has been shown to reduce the consequences of severe septic events [71], whereas the IL-33-mediated expansion of IL-4-producing basophils, upon high-dose immunoglobulin administration, has been shown to induce profound immunoregulatory effects [72]. The dichotomous nature of IL-33 has also led to the generation of conflicting data in the setting of intestinal inflammation. In fact, data generated on the spontaneous enteritis characterizing SAMP mice suggests a frank pathogenic role, while IL-33 KO mice undergoing DSS colitis develop a mixed response. In addition, chronic DSS colitis appears to be less severe after IL-33 administration [73]. In these studies, Groβ et al. induced both acute and chronic DSS colitis in balb/c mice and administered IL-33 to experimental animals using different protocols. When IL-33 was injected during the first cycle of DSS, colonic inflammation was more severe, with a dramatic increase in neutrophil infiltration; conversely, treating animals during the recovery phases of both acute and chronic DSS colitis decreased inflammatory scores and improved epithelial regeneration [73]. A different group reported partially overlapping results using a similar acute DSS protocol. Utilizing the acute DSS colitis model, Imaeda et al. administered IL-33 every 48 hours. In these studies, the authors reported increased inflammatory scores in IL-33-treated balb/c mice; however, when evaluating the epithelial layer, complete reversion of the goblet cell depletion characteristic of DSS colitis was observed [63]. This effect appeared to be mediated by the suppression of Notch ligand expression by SEMFs. In fact, the Notch pathway is a key regulator of epithelial cell differentiation, leading towards an absorptive phenotype [74]. As such, IL-33-mediated inhibition of the Notch pathway resulted in the maturation of epithelial cells towards a goblet cell phenotype, likely representing a protective response against harmful conditions [63]. Interestingly and along similar lines, IL-33 expression has been reported in SEMFs underlying ulcerated epithelia in UC and gastric ulcers [34, 75]. In addition, recent data suggest that IL-33 stimulation may increase gastric epithelial proliferation [76], whereas dramatic changes in the pattern of IL-33 expression in endothelial cells have been described during angiogenesis [77]. Overall, these data together strongly suggest that the IL-33/ST2 axis may be implicated in the maintenance of intestinal barrier function, wherein perturbations may likely play an important role in the development of chronic inflammatory conditions of the gut. As such, we can speculate that IL-33 may enhance bacterial clearance by inducing early granulocyte infiltration, promoting epithelial differentiation towards a mucus-secreting phenotype, and by facilitating wound healing. The redistribution/loss of ST2L within the epithelial compartment described during UC [33, 35] is consistent with the goblet cell depletion characterizing this particular disease and can also account for a defective wound healing process that can contribute to the chronicity of the inflammatory process.

**Figure 2:**
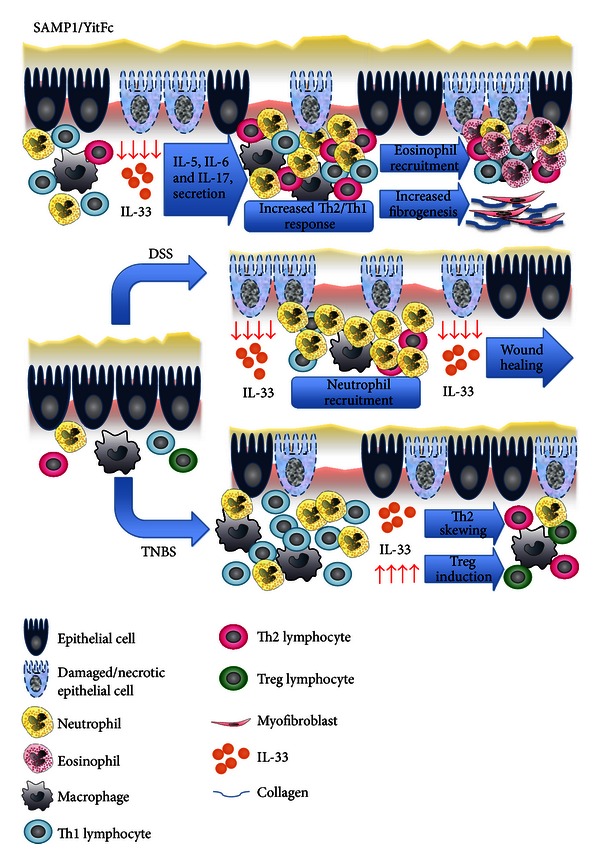
Role of IL-33 in murine models of intestinal inflammation. Experimental results obtained from SAMP mice that develop spontaneous Th1/Th2-driven enteritis suggest a pathogenic role for IL-33 at the onset of intestinal inflammation in this specific model. In fact, epithelial-derived IL-33 promotes the release of proinflammatory cytokines from LP immune cells, enhancing both Th1 and Th2 responses. Moreover, IL-33 induces the production of eotaxin-1 and eotaxin-2, which leads to eosinophil chemotaxis to the inflamed gut. In addition, IL-33 activates the expression of profibrotic genes, contributing to the development of intestinal fibrosis (upper panel). Different results are observed in chemically induced models of intestinal inflammation (middle and lower panels). During the onset of acute DSS-induced colitis, IL-33, likely released by necrotic/damaged epithelial cells, participates in the development of intestinal inflammation with a potent chemotactic effect on neutrophils, cells that play a pivotal role in this specific model of colitis (middle panel). Conversely, during the recovery phase of chronic DSS colitis, IL-33 appears to promote wound healing, inducing the restoration of epithelial barrier integrity. On the contrary, when IL-33 is administered to mice displaying a TNBS-induced colitis, which is mainly driven by a Th1 immune response, IL-33 shows an anti-inflammatory effect as a result of the skewing towards a Th2 immunophenotype and the potential induction of T regulatory cell activation (lower panel).

Data generated by Duan et al. using the Th1-driven TNBS-induced colitis, opposing to what was shown by Sedhom et al., suggested more immune-mediated/immunomodulatory properties of IL-33 [78]. Using this model, mice developed less severe colitis following intraperitoneal injections of IL-33, whereas anti-IL-33 antibody administration did not significantly affect intestinal inflammation. The anti-inflammatory effects of IL-33 appeared to be mediated by a decreased production of the prototypic Th1 cytokine, IFN*γ*, while the Th2 cytokines, IL-5 and IL-13, were found to be increased. The authors conclude that IL-33 has the ability to initiate Th1 to Th2 skewing. In addition, the authors also infer that IL-33 has the ability to promote tolerogenic dendritic cell development, which ultimately results in the expansion of the T regulatory cell population [78]. Thus, during Th1-driven inflammation, IL-33 may have the capability to modulate gut mucosal immune responses to a more Th2-driven phenotype and promote the expansion of regulatory cell types. The different functions of the IL-33/ST2 axis during intestinal inflammation in SAMP spontaneous enteritis and in the chemically induced DSS- and TNBS-induced colitis models are recapitulated in Figure 2. 

## 6. IL-33 As an Alarmin: A Possible Unifying Solution

Indeed, the IL-33/ST2 axis appears to be widely represented throughout the whole body, having different, and sometimes opposing, functions. The resulting balance between the differential effects appears to be straightforward in certain tissue/organ systems, such as the airways/lungs or the joints, where inflammation, in itself, is the major detrimental agent to cause pathology. In more complex systems, wherein mucosal immune responses interact with a large bacterial load and epithelial barrier integrity and function is essential to consider, such as that found in chronic intestinal inflammation, the final outcome of this intricate interplay is difficult to predict and may vary according to slightly modifications of the initial conditions. In fact, IL-33 appears to enhance intestinal inflammation in disease models, which are driven by Th2 and innate immune responses, such that observed in SAMP mice and the acute phase of DSS colitis, and possibly in UC patients. Conversely, IL-33's effects during a Th1-driven model, such as in TNBS colitis, may result in decreased intestinal inflammation mediated by cytokine and cell-mediated modulation of immune responses. On the other hand, emerging evidence suggests that IL-33 may have positive effects on epithelial repair and barrier function. High levels of IL-33 during acute inflammation are likely to worsen tissue damage, whereas they may enhance tissue repair during recovery, promoting wound healing and the restoration of the epithelial barrier, as shown in the DSS model. Thus, the initial features of the specific immune response and the timing of IL-33 administration/blockade may dictate the overall outcome of disease pathogenesis. 

The nuclear localization sequence in IL-33's primary structure suggested a possible role and function for this cytokine as an “alarmin,” that is, a protein released from dying/suffering cells as an extracellular sign of danger [79]. Consistent with this hypothesis is the fact that IL-33 is released by cells undergoing mechanical stress [80] and is cleaved by pro-apoptotic caspases into less active forms [10–12]. Moreover, data obtained on mononuclear cells confirm that IL-33 is overexpressed after TLR-2 and -4 stimulation, but it is released only when necrosis of these cells is induced [81]. On the same line, it has been shown that other danger signals, such as extracellular ATP, different pathogen-associated molecular patterns (PAMPs), and inflammasome activation, lead to the increased production of IL-33 in different cell types (i.e., glial cells, airway epithelial cells) [82–84]. Indeed, the “alarmin” paradigm may have the potential to reconcile how IL-33 possesses such a wide spectrum of effects in the gastrointestinal tract. That is, upon harmful stimuli, IL-33 is released by suffering epithelial barrier cells in order to recruit and activate immune cells and clear potential pathogens. At the same time, the presence of a defective and damaged epithelial barrier must be quickly repaired, and the need for prompt epithelial restitution and wound healing is promoted. 

Interestingly, IBD genetic studies have identified a few candidate susceptibility genes, encoding proteins that are pivotal for the maintenance of epithelial cell integrity, such as the endoplasmic reticulum stress protein, proteins related to the autophagy process, and structural proteins [1]. It is tempting to speculate that when epithelial function is severely impaired as a consequence of mutations of the aforementioned genes, suffering intestinal epithelial cells may release high levels of IL-33, activating a potentially detrimental immune response. At the same time, the loss/dysregulation of ST2L on intestinal epithelial cells in IBD may alter epithelial restoration. As such, further, more mechanistic investigation is warranted to dissect this complicated scenario, aiming to clarify the specific effects of the IL-33/ST2 axis, at different times as well as the contribution of different cellular sources, in order to elucidate the predominant role of this complex cytokine system in the pathogenesis of intestinal inflammation.
